# Supplementary material for: The Impact of Head-Up Tilt Sleeping on Orthostatic Tolerance: A Scoping Review
Source: Biology (Basel). 2023 Aug 9;12(8):1108. doi: 10.3390/biology12081108 (PMC10452159; doi:10.3390/biology12081108)
Supplement: Supplementary file 1 [file biology-12-01108-s001.zip › biology-2526707-supplementary.pdf]

Supplementary tables

Supplementary Table S1. Database search strategy consisting of part 1, terms to define sleeping in a head-up tilt position, and part 2, autonomic nervous system outcome measures.

Database	Search terms
**Pubmed**	Part 1<br>("Sleep*"[Mesh] OR sleep*[Title/Abstract] OR night*[Title/Abstract] OR nocturnal[Title/Abstract])<br>AND<br>(head up[Title/Abstract] OR head-up[Title/Abstract] OR tilt*[Title/Abstract] OR anti-trendelenburg[Title/Abstract] OR reverse trendelenburg[Title/Abstract] OR incline*[Title/Abstract] OR ((bed[Title/Abstract] OR head-of-bed[Title/Abstract]) AND<br>(elevat*[Title/Abstract])))<br>Part 2<br>("Hemodynamics"[Mesh] OR Hypotension"[Mesh] OR "Hypertension"[Mesh]<br>OR "Edema"[Mesh] OR "Urinary Tract Physiological Phenomena"[Mesh] OR "Nocturia"[Mesh] OR "Water-Electrolyte Balance"[Mesh] OR "Water-Electrolyte Imbalance"[Mesh] OR "Syncope"[Mesh] OR autonom*[Title/Abstract] OR blood pressure[Title/Abstract] OR hypotensi*[Title/Abstract] OR hypertensi*[Title/Abstract] OR syncope[Title/Abstract] OR Hemodynamic*[Title/Abstract] OR Haemodynamic*[Title/Abstract] OR Cardiac Output[Title/Abstract] OR Stroke Volume[Title/Abstract] OR Edema[Title/Abstract] OR Oedema[Title/Abstract] OR Nycturia[Title/Abstract] OR Nocturia[Title/Abstract] OR Vascular resistance[Title/Abstract] OR Vasodilatation[Title/Abstract] OR Vasoconstriction[Title/Abstract])
**Embase**	Part 1<br>((exp sleep/ OR<br>(sleep* OR night* OR nocturnal).ti,ab,kf.)<br>AND<br>(head up OR head-up OR tilt* OR anti-trendelenburg OR reverse Trendelenburg OR incline* OR ((head-of-bed OR bed) AND elevat*)).ti,ab,kf.)<br>Part 1<br>(cardiovascular function/ or blood vessel function/ or cardiovascular effect/ or cardiovascular performance/ or cardiovascular reflex/ or cardiovascular response/ or circulation/ or heart function/ or hemodynamics/ or exp abnormal blood pressure/ or edema/ or peripheral edema/ or nocturia/ or exp faintness/ or exp electrolyte disturbance/ or urinary tract function/ or bladder function/ or diuresis/ or kidney function/ or urine acidification/ or urine flow rate/ or autonomic neuropathy/)<br>OR (autonom* OR blood pressure OR hypotensi* OR hypertensi* OR syncope OR Hemodynamic* OR Haemodynamic* OR Cardiac Output OR Stroke Volume OR Edema OR Oedema OR Nycturia OR Nocturia OR Vascular resistance OR Vasodilatation OR Vasoconstriction).ti,ab,kf.)
**Cochrane**	Same as Pubmed
**Web of science**	Part 1<br>((TS=sleep* OR TS=night* OR TS=nocturnal)<br>AND<br>((TS="head up" OR TS=head-up OR TS=tilt* OR TS=anti-trendelenburg OR TS="reverse trendelenburg" OR TS=incline*) OR<br>((TS=bed OR TS=head-of-bed) AND TS=elevat*)))<br>Part 2<br>TS=Syncope OR TS=autonom* OR TS="blood pressure" OR TS=hypotensi* OR TS=hypertensi* OR TS=syncope OR TS=Hemodynamic* OR TS=Haemodynamic* OR TS="Cardiac Output" OR TS="Stroke Volume" OR TS=Edema OR TS=Oedema OR TS=Nycturia OR TS=Nocturia OR TS="Vascular resistance" OR TS=Vasodilatation OR TS=Vasoconstriction
